# Morphological and adhesive properties of *Klebsiella pneumoniae* biofilms

**DOI:** 10.14202/vetworld.2020.197-200

**Published:** 2020-01-28

**Authors:** Ekaterina Lenchenko, Dmitry Blumenkrants, Nadezhda Sachivkina, Nadezhda Shadrova, Alfia Ibragimova

**Affiliations:** 1 Department of Veterinary Medicine, Moscow State University of Food Production, Moscow, Russia; 2 Department of Microbiology and Virology, Medical Institute, Peoples’ Friendship University of Russia, Moscow, Russia; 3 Department of Veterinary Medicine, Agrarian Technological Institute, Peoples’ Friendship University of Russia, Moscow, Russia; 4 Department of Foreign Languages, Agrarian Technological Institute, Peoples’ Friendship University of Russia, Moscow, Russia

**Keywords:** adhesion coefficient, average adhesion index, biofilms, Gram-negative microflora, *Klebsiellapneumoniae*, optical density

## Abstract

**Background and Aim:**

The study of biofilm-forming ability of Gram-negative microflora has great practical importance for assessing the effectiveness of antibiotic therapy and finding new ways to diagnose and inhibit the growth of biofilms. This is because poor penetration of antibacterial drugs into the biofilm can lead to the selection of resistant strains and has a consequence evident by the occurrence of relapse of infection in animals. This study aimed to evaluate morphological and densitometric indicators of biofilm formation as well as adhesive properties of *Klebsiella* *pneumoniae*.

**Materials and Methods:**

*K. pneumoniae* was cultured at 37°C for 2-144 h *in vitro*. The specimens for optical microscopy were prepared by fixation with a 1:1 alcohol-ether mixture for 10 min and stained with a 0.5% solution of gentian violet for 2 min, and the optical density index was evaluated at a wavelength of 490 nm. Further, the adhesive properties of the microorganisms were determined at a concentration of 1 billion/ml and a suspension of ram erythrocytes at a concentration of 100 million/ml when cultured at 37°C for 24 h. Blood smears were prepared and stained with 0.5% gentian violet.

**Results:**

*K. pneumonia* cultured at 37°C after 24 h on the meat peptone agar formed large, convex, mucous, and white colonies (d=3.0-6.0 mm). With the growth in the meat and peptone broth, uniform turbidity of the medium was observed. Analyzing the optical density indices (density, D), it was found that *K. pneumoniae* were good producers of biofilms (D=0.528±0.31). Data for indicators of adhesive properties of *K. pneumoniae* were as follows: Average adhesion index, 4.56±0.14; adhesion coefficient, 1.07±0.52; and adhesion index, 4.26±0.07. The studied bacteria had high adhesive activity. A direct correlation dependence (R=0.94) of the optical density of biofilms (D≥0.514-0.551) and AAI (4.15±0.28-4.76±0.75) was established.

**Conclusion:**

This study has demonstrated that *K. pneumoniae* had high adhesive activity, was strong producer of biofilms, and the optical density of the sample exceeded the optical density of the control by more than 4 times.

## Introduction

Nosocomial infections are common and remain a major cause of mortality and morbidity worldwide. One of the main causative agents of these infections is *Klebsiella pneumoniae*, a bacterium with a high level of antibiotic resistance. Therefore, the investigation of these microorganisms aAnd their susceptibility to antibiotics is essential [1,2]. Most diseases related to animal digestive and respiratory systems are caused by *K. pneumoniae*. These diseases (such as gastritis, enteritis, hepatitis, and pneumonia) can be acute, subacute, as well as chronic; hence, they lead to increased animal mortality [3]. When the immunity of infected animal decreases, the virulence of the pathogen increases, which leads to an increase in the effect of toxins on the intestinal mucosa, overpowering of local defense factors, inflammation, and dysbacteriosis. By entering the bloodstream, *Klebsiella* causes the development of septicemia, which can be life-threatening.

*Klebsiella* is a Gram-negative, non-motile, and rod-shaped bacteria. The bacterium has a capsule; it is resistant to the environment and action of disinfectants as well as many antibiotics, which makes it lethal. It has a complex antigenic structure and contains capsular and somatic antigens and endotoxin; some strains can produce exotoxin. These microorganisms can cause pneumonia, acute intestinal infections, urogenital infections, conjunctivitis, meningitis, and sepsis in lambs [4]. *Klebsiella* infections can also develop as a secondary infection against the background of infection by viruses, which can also lead to an increase in the number of deaths.

For pathogenic microorganisms to survive in the conditions of the biotope, they must acquire certain properties, including the formation of biofilms [5-7]. As a result, they subsequently acquire the ability to resist the factors of natural forces such as some macroorganisms and antimicrobial agents of various origins [8]. From this point of view, elucidation of the mechanism by which these microorganisms acquire the ability to produce biofilms and adhere to animal cells is very important.

Hence, this study aimed to evaluate the morphological and densitometric indicators of *K. pneumoniae* biofilms, and identify its adhesive properties.

## Materials and Methods

### Ethical approval

This work with the certified strain of *K. pneumoniae* does not require authorization from the Ethics Committee.

### Strain

The experiments used a certified strain of *K. pneumoniae* from the collection of the state research institute for standardization and control of medical biological preparations named after L.A. Tarasevich (Moscow). This microorganism was isolated from rabbits of “Soviet chinchilla” breed. Most products made with rabbit fur are from the skins of animals of this variety in Russia.

### Optical density measurement

The morphology of the biofilms was evaluated by culturing the bacteria at 37°C for 2, 4, 6, 24, 48, 72, and 144 h and specimens for microscopic studies were prepared from cultures. The specimens for optical microscopy were prepared by fixation in a 1:1 alcohol-ether mixture for 10 min and stained with a 0.5% solution of gentian violet, and allowed to stay for 2 min [3]. To obtain representative information, random sampling of the field of view under the microscope (H604T Trinocular Unico, USA) was performed.

The optical density of the biofilms was determined based on the degree of binding of violet crystal at a wavelength of 490 nm, using the “*Immunochem-2100*” microplate photometric analyzer (HTI, USA). Values were determined in triplicates and classified as follows:

1. Weak biofilm producers (*Density of the sample* – D_s_): These were samples with an optical density <2 times the optical density of the control (*Density of the control* – D_c_) (D≤0.197)
2. Moderate biofilm producers: Here, the optical density of the sample was 2-4 times more than the optical density of the control (D=0.279-0.571)
3. Strong producers of biofilms were samples with an optical density that was 4 times more than that of the control (D=0.699-1.510) [9,10].

### Adhesive property testing

The culture of *K. pneumoniae* at a concentration of 1 billion/ml and a suspension of ram erythrocytes at a concentration of 100 million/ml were cultured at 37°C for 24 h, blood smears were prepared and stained with 0.5% solution of gentian violet [11].

### Statistical analysis

The experimental data were processed using descriptive and inferential statistics. Means and standard deviations of the optical densities and adhesive properties were calculated using Microsoft Excel. Difference between means of samples and that of the control was determined using the Student’s t-test, and statistical significance of the differences was set at p≤0.05.

## Results and Discussion

The study revealed that microorganism cultures of *K. pneumoniae* at 37°C after 24 h on the meat peptone agar formed large, convex mucous, and white colonies, d=3.0-6.0 mm. With the growth in the meat and peptone broth, uniform turbidity of the medium was observed. *K. pneumoniae* are facultative anaerobic bacteria; the growth of these bacteria was studied under aerobic and anaerobic conditions.

Microorganisms of the genus *Klebsiella* utilized glucose, sodium citrate while producing acetylmethylcarbinol, fermented inositol, and hydrolyzed urea, however, it did not form indole and hydrogen sulfide.

**Figure-1:**
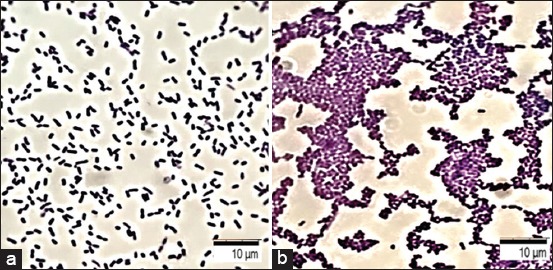
Formation of bacterial biofilms of *Klebsiella pneumoniae*: (a) Reversible adhesion of vegetative forms of bacteria having a typical shape and size (0.3-0.6×0.6-1.0); (b) the formation of a monolayer of bacteria – a diffuse layer of bacterial cells; coloring with a gentian violet; optical microscopy, 100×.

The morphology of the biofilms was studied *in vitro* after 2-8 h of cultivation of the microorganisms. Reversible adhesion of vegetative forms of bacteria was observed, as well as deformation of the cell wall as a result of the attachment of an abiotic substrate coverslip to the surface, as shown in Figure-1a. After 10-12 h of cultivation, irreversible adhesion was observed on the plate. Under the microscope, blue-stained bacterial cells were combined into short chains while in some areas, long strands surrounded by an intercellular matrix of different color intensities were observed.

In addition, after about 24-36 h of cultivation, the formation of a monolayer of bacteria with a diffuse layer of bacterial cells was observed (Figure-1b). The process of coaggregation ensured the formation of microcolonies in individual areas by the formation of intercellular bonds. Densely packed cell structures of various sizes and shapes connected by an intercellular matrix were identified as well. Moreover, formation of a mature biofilm was observed due to the fusion of microcolonies with the subsequent formation of clusters after 48-72 h of cultivation. Rounded pores and tubules containing fluid and surrounded by membrane structures were detected on the edges or borders of cluster formation. The mechanical stability was ensured by exocellular substances identified on the surface of the biofilms in the form of polymer networks, consisting mainly of polysaccharides.

Two types of intercellular contacts were observed: The first was direct interaction, followed by the formation of clusters mediated by the interconnection matrix. This confirmed the active production of a substance filling the intercellular space after adhesion to the surface by the bacteria. Then, due to the cells attached to the substrate and the intercellular matrix, a diffuse layer was formed on the surface of the abiotic substrate; the cells were arranged in groups in the form of a monolayer united by a common intercellular matrix.

After 72-144 h of cultivation, there was the dispersion of colonies. In some areas, destructive processes of the intercellular matrix, accompanied by the separation of cells that retained the ability to adhere and form new microcolonies were identified.

Analyzing the optical density indices (*Density*, D), it was found that *K. pneumoniae* were strong producers of biofilms, the optical density of the sample (*Density of the sample* – D_s_) exceeded the optical density of the control (*Density of the control* – D_c_) by more than 4 times (D=0.528±0.31).

**Figure-2:**
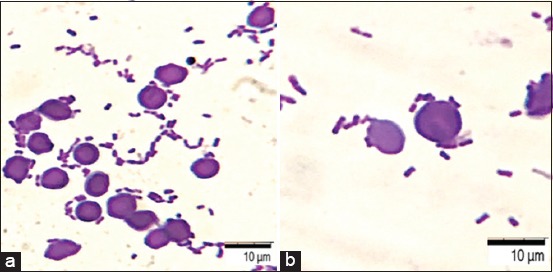
Adhesion to red blood cells, 30 min, *Klebsiella pneumoniae*. Staining with a gentian violet, optical microscopy: (a) 100×; (b) 200×.

During optical microscopy of the preparations, three following indicators were taken into account: 1 - Average adhesion index (AAI), which is the average number of microorganisms attached to the surface of a single red blood cell; 2 - adhesion coefficient (AC), percentage of red blood cells having bacteria on the surface; 3 - microorganism adhesion index (AI) known as ratio of AAI and AC (Figure-2).

Indicators of adhesive properties of bacteria *K. pneumoniae* amounted to the following: AAI = 4.56±0.14; AC =1.07±0.52; and AI = 4.26±0.07. Depending on the AI values, all bacteria can be considered: Non-adhesive (AI=1.00-1.75); low adhesive (AI=1.76-2.49); medium adhesive (AI=2.50-3.99); and highly adhesive (AI ≥4.00) categories. The studied bacteria had high adhesive activity. A direct correlation dependence (R=0.94) of the optical density of biofilms (D≥0.514-0.551) and AAI (4.15±0.28-4.76±0.75) were established.

This study found that the identification of the pathogenic properties of microorganisms was confirmed by adhesive properties. Earlier studies have shown that high adhesive potential is one of the key factors in the formation of a biofilm architectonics, characterized by an increase in optical density, causing multiple drug resistance [12-15]. Furthermore, 57.1% of *K. pneumoniae* strains were strong biofilm producers, as observed by another study [16]. A correlation was established between the ability of *K. pneumoniae* from a biofilm and a profile of resistance to antibacterial drugs: Resistant strains were assigned to strong producers of biofilms (91.07 %) [17].

*K. pneumoniae* isolates were classified as multidrug-resistant bacteria. Resistance to colistin, doxycycline, ciprofloxacin, and enrofloxacin ranged from 18.2 to 90.9%. Genes with an extended spectrum of beta-lactamases were detected in 93.4% of isolates from nasal swabs, and two genetic virulence factors were found in 100.0% of strains [18].

Inhibition of *K. pneumoniae* biofilm was observed when papain was exposed to antibiotic-resistant strains (10.6-56.2%) and increased (21.4-59.0%) at a concentration of 100 mg/ml, which indicates the potential use of the enzyme in combination with antibacterial drugs [19-21].

The stability of the formation of *K. pneumoniae* biofilms changed on exposure to 3-methyl-2 (5H)-furanone and 2′-hydroxycinnamic acid, inhibiting optical density by 67.38% and 65.06%, respectively. The formed biofilms were more susceptible to gentamicin [22-24].

## Conclusion

This study provides data on the morphological and densitometric indicators of biofilms, as well as the adhesive properties of *K. pneumonia*. We found that the microorganisms have high adhesive activity and are strong producers of biofilms. The optical density of the samples exceeded the optical density of the control by more than 4 times. A direct correlation dependence (r=0.94) of the indicators of the optical density of biofilms and the degree of adhesion of the bacteria was established. The high adhesive potential is one of the key factors in the formation of the biofilm architectonics, characterized by an increase in optical density, and causing multiple drug resistance.

## Authors’ Contributions

EL and DB had the original idea for the study and carried out the design. NSh collected the samples. AI was responsible for data analysis and data cleaning. EL and NSa drafted the manuscript. The final draft manuscript was revised by all authors. All authors edited, read, and approved the final manuscript.
